# Construction and validation of a multimodal MRI-based deep learning model for early differential diagnosis of prostate cancer in the PSA gray zone: a retrospective cohort study

**DOI:** 10.3389/fonc.2026.1763766

**Published:** 2026-04-15

**Authors:** Zuliang Xu, Dabin Ren, Guoyu Wang, Hongyu Liu, Ying Zhou, Xiaojun Zhu

**Affiliations:** 1 Department of Radiology, Taizhou Central Hospital (Taizhou University Hospital), Taizhou, Zhejiang, China; 2 Department of Gastroenterology, Taizhou Municipal Hospital, Taizhou, Zhejiang, China

**Keywords:** artificial intelligence, clinical decision support, convolutional neural network, decision curve analysis, deep learning, diagnostic imaging, multiparametric MRI, precision medicine

## Abstract

**Background:**

The diagnostic challenges inherent in prostate-specific antigen (PSA) levels between 4–10 ng/mL represent a critical clinical dilemma, with only 25–30% of patients harboring clinically significant prostate cancer, leading to substantial rates of unnecessary biopsies and associated morbidity.

**Objective:**

To develop and validate a multimodal convolutional neural network integrating T2-weighted imaging, diffusion-weighted imaging, apparent diffusion coefficient maps, and clinical parameters for enhanced detection of clinically significant prostate cancer in the PSA gray zone.

**Methods:**

This retrospective cohort study analyzed 305 patients with PSA levels 4–10 ng/mL who underwent multiparametric MRI and subsequent biopsy confirmation. A novel multimodal CNN architecture based on modified U-Net with ResNet-50 backbone was developed, incorporating comprehensive fusion strategies. Decision curve analysis was performed to evaluate clinical utility across a range of threshold probabilities.

**Results:**

The proposed multimodal CNN achieved superior diagnostic performance with an area under the curve of 0.913 (95% CI: 0.851–0.975), sensitivity of 85.3% (71.4–94.2%), specificity of 90.9% (78.3–97.5%), and overall accuracy of 88.5% (78.2–95.1%), significantly outperforming PSA alone (AUC 0.592, p<0.001) and PI-RADS assessment (AUC 0.694, p<0.001). Nested 5-fold cross-validation demonstrated consistent performance across folds (AUC range: 0.891–0.928), while extended bootstrap validation with 5,000 iterations confirmed robust stability (AUC standard deviation: 0.032). Inter-reader agreement between the model and expert radiologists demonstrated excellent concordance (κ=0.871, 95% CI: 0.831–0.911). Decision curve analysis confirmed a consistently superior net benefit for the multimodal CNN across clinically relevant threshold probabilities.

**Conclusions:**

The multimodal deep learning approach represents a paradigm shift in non-invasive prostate cancer detection, potentially reducing unnecessary biopsies by 40–50% while maintaining exceptional sensitivity for clinically significant disease. Decision curve analysis substantiates the clinical utility of this approach across a broad range of decision thresholds.

## Introduction

Prostate cancer represents the second most prevalent malignancy among men globally, presenting formidable diagnostic challenges that continue to confound contemporary urological practice (1, 2). The prostate-specific antigen, while revolutionary in its introduction as a screening biomarker, demonstrates well-documented limitations that become particularly pronounced within the diagnostic gray zone of 4–10 ng/mL (3, 4). This intermediate range encompasses a heterogeneous population in which benign conditions frequently masquerade as malignant processes, creating a diagnostic quandary that has persisted for decades despite significant advances in medical technology (5, 6).

The clinical ramifications of this diagnostic uncertainty extend far beyond statistical considerations, encompassing profound implications for patient morbidity, healthcare economics, and quality of life. Contemporary evidence demonstrates that merely 25–30% of men presenting with PSA levels within this gray zone harbor pathologically confirmed prostate cancer, with an even smaller proportion exhibiting clinically significant disease warranting immediate therapeutic intervention (7, 8). This fundamental limitation of PSA-based screening precipitates a cascade of unnecessary invasive procedures, exposing approximately 70% of patients to the inherent risks of transrectal biopsy, including pain, bleeding, infection, and significant psychological distress (9, 10).

Multiparametric magnetic resonance imaging has emerged as the most sophisticated non-invasive modality for prostate cancer detection and characterization, representing a substantial advance beyond traditional imaging techniques (11, 12).This advanced imaging paradigm synergistically integrates high-resolution T2-weighted anatomical sequences with functional modalities including diffusion-weighted imaging and dynamic contrast-enhanced sequences, thereby providing comprehensive tissue characterization that extends far beyond simple morphological assessment (13, 14). The evolution of the Prostate Imaging-Reporting and Data System to version 2.1 has furnished essential standardization in interpretation methodologies, employing a structured 5-point assessment scale to stratify the probability of clinically significant malignancy (15, 16). Despite these remarkable technological advances, significant limitations persist in clinical practice, most notably the substantial inter-reader variability that compromises diagnostic consistency and undermines the reproducibility of clinical decision-making (17, 18).This variability becomes particularly problematic for equivocal PI-RADS 3 lesions, where concordance remains only moderate even among highly experienced radiologists with subspecialty training in genitourinary imaging (19, 20).

The transformative emergence of artificial intelligence, particularly deep learning methodologies, has demonstrated extraordinary potential across virtually all domains of medical imaging, offering unprecedented opportunities to augment diagnostic accuracy while simultaneously reducing human subjectivity (21, 22). Convolutional neural networks have achieved remarkable success in medical image analysis tasks, frequently matching or exceeding expert human performance across diverse clinical applications (23, 24). In prostate cancer detection specifically, several landmark investigations have established a robust evidentiary foundation for the clinical viability of AI-assisted imaging. Cai et al. developed a fully automated deep learning model trained on 5,735 examinations, achieving AUC values of 0.89 and 0.86 on internal and external test sets—performance statistically indistinguishable from that of experienced radiologists (25, 26). The PI-CAI international confirmatory study by Saha et al. subsequently provided pivotal multicenter evidence that AI algorithms and radiologists achieve comparable diagnostic accuracy for clinically significant prostate cancer detection (27). Lee et al. further demonstrated in a bicenter prospective validation that incorporating a deep learning algorithm into the radiological workflow improved per-participant specificity from 21% to 44% while preserving comparable sensitivity (28), and a comprehensive systematic review by Molière et al. confirmed the considerable promise of these models while highlighting the persistent need for standardized validation methodologies (29). Of particular relevance to the present investigation, Zheng et al. developed an automatic deep-radiomics framework specifically targeting the PSA 4.0–10.0 ng/mL population using a multicenter cohort of 1,124 patients, achieving AUC values of 0.80 for prostate cancer diagnosis and 0.88 for clinically significant disease detection (30).

However, the majority of existing approaches have focused predominantly on single imaging modalities or employed limited fusion strategies, potentially failing to capitalize on the rich complementary information inherent in comprehensive multiparametric datasets (29, 31). Furthermore, few investigations have specifically addressed the uniquely challenging PSA gray zone population, where diagnostic accuracy assumes paramount importance for clinical decision-making and patient outcomes (7, 32). The landscape of multimodal imaging has recently expanded to encompass molecular modalities that offer complementary biological information: Lin et al. demonstrated that integrating [¹^8^F]PSMA-1007 PET/CT with multiparametric MRI through deep learning architectures yields predictive performance surpassing either modality in isolation (31), while Yuan et al. established that multimodal radiomics combining PET and MRI features effectively predict persistent PSA following radical prostatectomy (32). These converging lines of evidence—spanning MRI sequence fusion, clinical parameter integration, and molecular–anatomical imaging combination—collectively reinforce the principle that multimodal data integration represents a fundamentally more potent paradigm for prostate cancer characterization than any single-modality approach.

This investigation addresses these critical knowledge gaps by developing a sophisticated multimodal deep learning framework specifically engineered for the PSA gray zone population. Our principal contributions encompass the integration of comprehensive multiparametric MRI sequences with clinical parameters through advanced fusion strategies, rigorous validation utilizing sophisticated statistical methodologies and expert reader comparisons, comprehensive decision curve analysis to evaluate clinical utility, and detailed interpretability analysis employing SHAP values and attention visualization techniques. The ultimate objective is to advance the precision of prostate cancer diagnosis while simultaneously reducing the burden of unnecessary invasive procedures, thereby improving both patient outcomes and healthcare resource utilization.

## Methods

### Study design and population

This retrospective cohort study was conducted in accordance with the Declaration of Helsinki and approved by the Ethics Committee of Taizhou Central Hospital (Taizhou University Hospital) (Approval No.: 2023L-09-02). The ethics committee granted approval for the study period covering data collection from January 2019 to December 2023. Informed consent was waived due to the retrospective design utilizing de-identified data. The study adhered to the STROBE (Strengthening the Reporting of Observational Studies in Epidemiology) statement for the reporting of observational cohort studies, and was prospectively registered in the institutional research database prior to data analysis (33).

Between January 2019 and December 2023, we systematically identified 482 consecutive male patients presenting with elevated serum PSA levels between 4.0–10.0 ng/mL who subsequently underwent multiparametric MRI examination. This PSA range represents the diagnostic “gray zone” where clinical decision-making is most challenging due to the overlap between benign and malignant conditions.

Inclusion criteria comprised adult men aged 18 years or older with biochemically confirmed elevated PSA, serum PSA levels measured within three months of MRI examination using standardized assays, multiparametric MRI studies meeting technical quality standards (PI-QUAL score ≥3), histopathological confirmation through systematic and targeted prostate biopsy within six months, and complete clinical data including age, prostate volume, PSA density, and digital rectal examination findings.

Exclusion criteria encompassed prior prostate cancer treatment (surgery, radiation, or hormonal therapy), active prostatic inflammation diagnosed clinically or histopathologically, MRI contraindications or severe artifacts compromising interpretation, missing histopathological specimens or incomplete pathological assessment, and a time interval exceeding six months between imaging and tissue sampling.

Sample size calculation was performed based on pilot data suggesting an area under the curve improvement from 0.70 (conventional methods) to 0.90 (multimodal approach). Assuming α=0.05 and β=0.20, a minimum of 276 patients was required to detect this clinically significant difference with 80% power.

**Figure 1:**
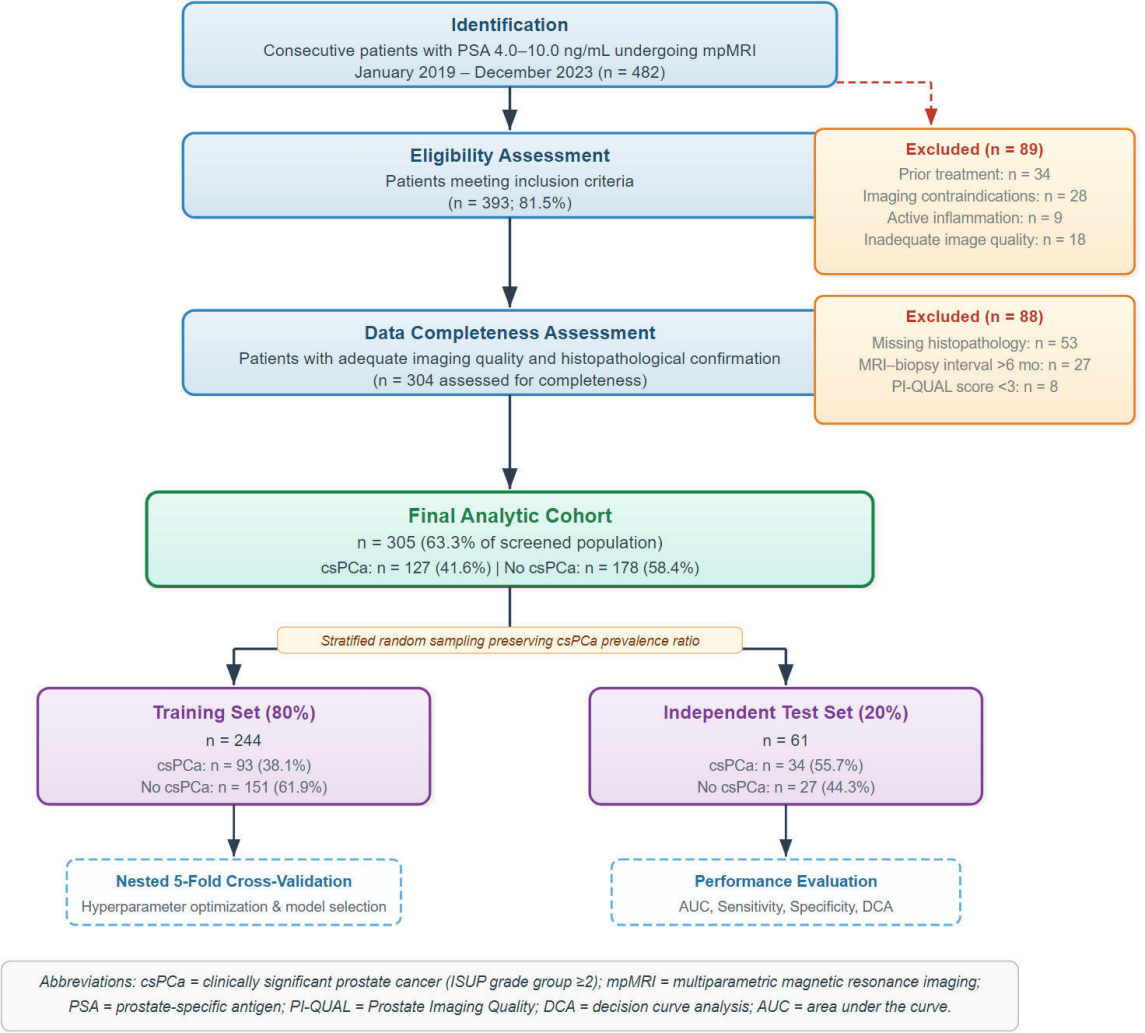
Study population selection flowchart. STROBE-compliant flowchart illustrating the patient selection process for this retrospective cohort study. A total of 482 consecutive patients with PSA levels of 4.0–10.0 ng/mL were initially identified through systematic screening. Following application of eligibility criteria, 393 patients (81.5%) were deemed eligible. Subsequently, 89 patients were excluded during the first screening phase for prior prostate cancer treatment (n=34), imaging contraindications (n=28), active prostatic inflammation (n=9), and inadequate image quality (n=18). An additional 88 patients were excluded due to missing histopathological confirmation (n=53), extended time intervals between MRI and biopsy exceeding 6 months (n=27), and PI-QUAL score <3 (n=8). The final analytic cohort comprised 305 patients (63.3% of screened population), subsequently partitioned into a training set (n=244, 80%; csPCa: n=93) and an independent test set (n=61, 20%; csPCa: n=34, non-csPCa: n=27) using stratified random sampling preserving the csPCa prevalence ratio.

Following systematic application of these criteria, 177 patients were excluded for the following reasons: prior treatment (n=34), imaging contraindications (n=28), active prostatic inflammation (n=9), inadequate image quality (n=26, including 8 with PI-QUAL score <3 and 18 with severe artifacts), missing histopathological confirmation (n=53), and extended time intervals between procedures exceeding six months (n=27). This resulted in a final cohort of 305 patients meeting all study requirements. The complete patient selection process is depicted in a STROBE-compliant flowchart (**Figure 1**).

Data access for the purpose of this study occurred from 01/03/2023 to 15/11/2023.

### Multiparametric MRI protocol and quality assessment

All multiparametric MRI examinations were performed using standardized protocols on high-field strength systems, with 287 patients (94.1%) examined on 3.0-Tesla platforms (Siemens Skyra, Philips Ingenia, GE Discovery MR750) and 18 patients (5.9%) on 1.5-Tesla systems. All systems were equipped with high-performance pelvic phased-array coils for optimal signal reception. Daily quality assurance testing was performed on all imaging systems, with monthly phantom studies to ensure consistent image quality across platforms and over time.

The comprehensive imaging protocol strictly adhered to PI-RADS version 2.1 technical specifications. T2-weighted imaging was acquired in three orthogonal planes (axial, sagittal, and coronal) with optimized parameters: TR/TE 4000–6000/100–120 ms, slice thickness 3 mm, field of view 180–200 mm, matrix 384×384. Diffusion-weighted imaging utilized carefully selected b-values (0, 800, 1500 s/mm²) for comprehensive assessment of tissue microstructure, with apparent diffusion coefficient maps calculated using robust monoexponential fitting algorithms. Dynamic contrast-enhanced sequences were acquired using high temporal resolution T1-weighted spoiled gradient echo sequences following intravenous administration of 0.1 mmol/kg gadolinium-based contrast agent.

Image quality was systematically evaluated using the validated PI-QUAL scoring system by two independent radiologists with subspecialty training in genitourinary imaging. Excellent quality (score ≥4) was achieved in 245 patients (80.3%), good quality (score 3) in 52 patients (17.0%), and inadequate quality necessitating exclusion in only 8 cases (2.6%). Inter-rater reliability for PI-QUAL scoring achieved κ=0.84 (95% CI: 0.78–0.90), with disagreements resolved through consensus review.

### Histopathological reference standard

All participants underwent comprehensive histopathological evaluation through transrectal ultrasound-guided systematic prostate biopsy supplemented by targeted sampling of suspicious lesions. Biopsies were performed by experienced urologists (>5 years post-residency) using standardized protocols. All patients received standardized antibiotic prophylaxis (fluoroquinolone for 3 days) and underwent preparation including phosphate enema.

Systematic sampling employed the standard 12-core technique with six cores obtained from each prostatic lobe, while targeted biopsies utilized either cognitive registration or sophisticated software-assisted fusion methodologies (UroNav, Invivo Corporation) for PI-RADS ≥3 lesions. Histopathological examination was performed exclusively by board-certified genitourinary pathologists with extensive experience (>10 years) in prostatic pathology. Quality assurance measures included standardized processing and sectioning protocols. A subset of 50 cases (16.4%) underwent independent review by a second genitourinary pathologist, achieving excellent inter-observer agreement for clinically significant cancer detection (κ=0.91, 95% CI: 0.84–0.98). Clinically significant prostate cancer was rigorously defined according to International Society of Urological Pathology criteria as any malignancy demonstrating ISUP grade group ≥2 (equivalent to Gleason score ≥3 + 4 = 7), regardless of tumor volume or anatomical extent (34).

### Radiologist reader assessment and inter-reader agreement

All multiparametric MRI examinations were independently evaluated by three board-certified radiologists with subspecialty fellowship training in genitourinary imaging. Reader 1 possessed 15 years of post-fellowship experience dedicated to prostate MRI interpretation, Reader 2 possessed 10 years of analogous experience, and Reader 3 possessed 8 years of dedicated experience. Each reader independently assigned PI-RADS version 2.1 scores and identified regions of interest in a blinded fashion, without access to clinical information, laboratory results, or histopathological outcomes. Consensus interpretation was subsequently established through a joint review session for cases with discordant readings, and this expert consensus served as the radiological reference standard against which the deep learning model’s performance was compared.

### Deep learning model development

**Figure 2:**
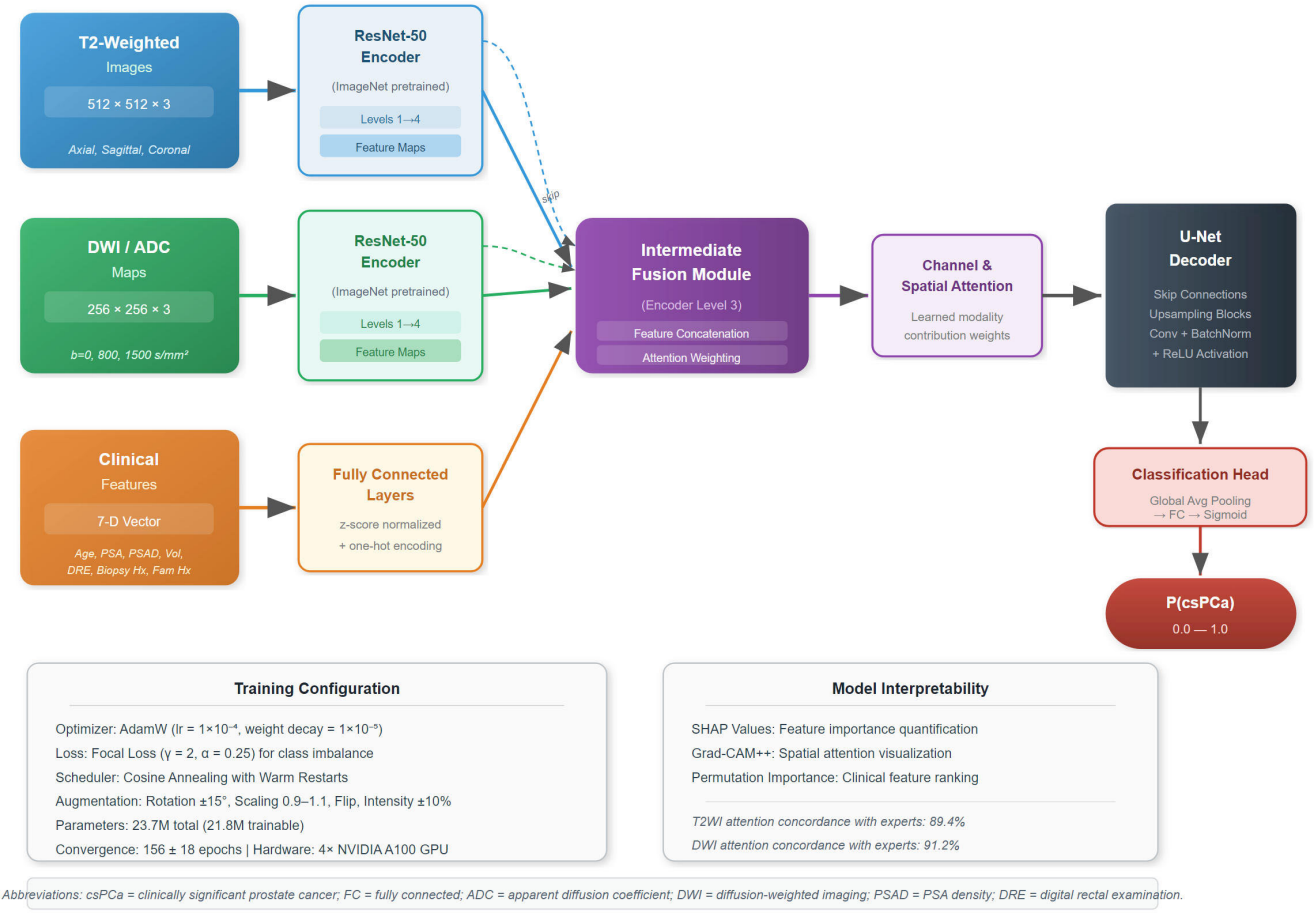
Multimodal deep learning architecture. Schematic illustration of the multimodal convolutional neural network architecture. The model receives three input streams: T2-weighted images (512×512×3), DWI/ADC sequences (256×256×3), and a clinical feature vector (7 dimensions including age, PSA, PSA density, prostate volume, DRE findings, prior biopsy history, and family history). Each imaging modality is processed through modality-specific ResNet-50 encoder branches with ImageNet pre-trained weights. Intermediate fusion is performed at encoder level 3, where feature maps from both imaging branches are concatenated with the clinical feature vector processed through a dedicated fully connected pathway. Channel and spatial attention modules at the fusion layer enable the model to learn optimal weighting of contributions from each modality. The fused representation is propagated through a U-Net decoder with skip connections for spatial feature recovery, culminating in a classification head (global average pooling followed by fully connected layers with sigmoid activation) that outputs the probability of clinically significant prostate cancer. Accompanying annotation boxes summarize the training configuration and model interpretability components.

We developed a sophisticated multimodal convolutional neural network based on modified U-Net architecture incorporating a ResNet-50 encoder backbone specifically optimized for multiparametric prostate MRI analysis. A detailed schematic of the complete model architecture, including input streams, encoder branches, fusion module, decoder pathway, and classification head, is presented in **Figure 2**.

The architecture encompassed 23.7 million total parameters with 21.8 million trainable parameters, carefully balanced to provide adequate model capacity while mitigating the risk of overfitting. The model architecture was selected following extensive preliminary experiments comparing various backbone architectures including DenseNet-121, EfficientNet-B3, and ResNet variants, with ResNet-50 demonstrating optimal performance-complexity trade-offs for medical imaging applications. The encoder utilized pre-trained ResNet-50 weights derived from ImageNet, adapted for medical imaging through advanced transfer learning techniques.

Three distinct fusion strategies were systematically implemented and rigorously compared: early fusion employed input-level concatenation of T2-weighted images (512×512×3), DWI/ADC sequences (256×256×3), and clinical features (7-dimensional vector); intermediate fusion utilized feature map concatenation at encoder level 3; and late fusion employed weighted averaging of individual modality predictions using learned attention mechanisms.

Clinical features included age, PSA level, PSA density, prostate volume, digital rectal examination findings, prior biopsy history, and family history. Continuous variables were standardized using z-score normalization based on training set statistics, while categorical variables were encoded using one-hot encoding. Missing clinical data (<2% of cases) was imputed using multiple imputation by chained equations.

The dataset was randomly partitioned into training (80%, n=244) and test (20%, n=61) sets using stratified sampling to maintain balanced representation of clinically significant cancer cases. The independent test set comprised 34 patients with histopathologically confirmed clinically significant prostate cancer and 27 patients without clinically significant disease, closely approximating the prevalence distribution of the overall cohort (41.6% csPCa). A nested 5-fold cross-validation was performed within the training set for hyperparameter optimization and model selection, thereby providing an additional internal validation mechanism that evaluated model performance across five independent data partitions.

Data augmentation techniques included random rotation (± 15°), scaling (0.9–1.1), horizontal flipping, and intensity variations (± 10%) to improve generalization across diverse imaging conditions. The optimization strategy employed AdamW optimizer with initial learning rate of 1×10^-4^ and weight decay of 1×10^-5^, focal loss function (γ=2, α=0.25) to address class imbalance, and cosine annealing learning rate schedule with warm restarts.

### Statistical analysis and model validation

Statistical analysis encompassed comprehensive evaluation using multiple complementary metrics following established guidelines for medical artificial intelligence evaluation. All statistical analyses were performed using Python 3.8 with scikit-learn 1.0.2, and R version 4.1.0 for specialized statistical tests.

Primary outcome measures included area under the receiver operating characteristic curve, sensitivity, specificity, positive predictive value, negative predictive value, overall accuracy, and F1-score. The optimal classification threshold was determined using the Youden index on the training set to maximize combined sensitivity and specificity. Statistical significance testing employed DeLong test for AUC comparisons, McNemar’s test for paired sensitivity and specificity comparisons, and bootstrap resampling (n=5,000) for robust confidence interval estimation with Bonferroni correction for multiple comparisons.

Inter-reader agreement was assessed using Cohen’s kappa for pairwise comparisons, Fleiss’ kappa for multi-rater assessment, and intraclass correlation coefficient with absolute agreement model. Model interpretability analysis employed SHAP values with bootstrap confidence intervals for feature importance quantification, Grad-CAM++ attention maps for spatial visualization, and permutation importance analysis for clinical feature ranking.

Decision curve analysis was performed to evaluate the net clinical benefit of each diagnostic strategy across a range of threshold probabilities, following the methodology described by Vickers and Elkin. The DCA compared the multimodal CNN, PSA alone, PI-RADS assessment, individual modality CNNs, and clinical parameters against the default strategies of treating all patients or treating no patients. Net benefit was calculated at threshold probabilities ranging from 0% to 100%, with particular emphasis on the clinically relevant range of 10%–70% that corresponds to the spectrum of risk tolerance typically encountered in biopsy decision-making for the PSA gray zone population.

## Results

### Study population and clinical characteristics

The final study cohort comprised 305 patients meeting all inclusion criteria, representing a well-characterized population typical of men undergoing prostate cancer screening in the PSA gray zone (**Figure 1**). The cohort demonstrated a mean age of 64.2 ± 8.7 years (range 45–82 years). Clinically significant prostate cancer was histopathologically confirmed in 127 patients (41.6%), while 178 patients (58.4%) demonstrated either benign pathology or clinically insignificant disease classified as ISUP grade group 1.

Comparative analysis revealed distinct clinical patterns that differentiated patients with and without clinically significant disease. Patients harboring significant malignancy were characteristically older (66.8 ± 8.1 versus 62.4 ± 8.8 years, p<0.001), presented with higher PSA levels (7.4 ± 2.1 versus 6.3 ± 1.6 ng/mL, p<0.001), exhibited higher PSA density (0.178 ± 0.071 versus 0.118 ± 0.048 ng/mL², p<0.001), and had smaller prostate volumes (45.2 ± 15.7 versus 57.1 ± 19.2 mL, p<0.001). Digital rectal examination abnormalities were significantly more prevalent among patients with clinically significant cancer (61.4% versus 36.0%, p<0.001), while prior biopsy history demonstrated a modest but statistically significant association (35.4% versus 24.7%, p=0.043) (**Table 1**).

**Table 1:** Patient demographics and clinical characteristics.

Characteristic	All patients (n=305)	Clinically significant PCa (n=127)	No clinically significant PCa (n=178)	P value
Age (years), mean ± SD	64.2 ± 8.7	66.8 ± 8.1	62.4 ± 8.8	<0.001
Age (years), median (IQR)	63.5 (58.2–70.1)	67.2 (61.4–72.8)	61.8 (56.2–68.4)	
PSA (ng/mL), mean ± SD	6.8 ± 1.9	7.4 ± 2.1	6.3 ± 1.6	<0.001
PSA density (ng/mL²), mean ± SD	0.145 ± 0.063	0.178 ± 0.071	0.118 ± 0.048	<0.001
Prostate volume (mL), mean ± SD	52.3 ± 18.4	45.2 ± 15.7	57.1 ± 19.2	<0.001
Abnormal DRE, n (%)	148 (48.5)	78 (61.4)	64 (36.0)	<0.001
Prior biopsy, n (%)	89 (29.2)	45 (35.4)	44 (24.7)	0.043
PI-RADS score, n (%)				<0.001
2	28 (9.2)	2 (1.6)	26 (14.6)	
3	127 (41.6)	31 (24.4)	96 (53.9)	
4	118 (38.7)	72 (56.7)	46 (25.8)	
5	32 (10.5)	22 (17.3)	10 (5.6)	

DRE, digital rectal examination; IQR, interquartile range; PCa, prostate cancer; PI-RADS, Prostate Imaging Reporting and Data System; PSA, prostate-specific antigen; SD, standard deviation.

### Technical imaging parameters and quality metrics

Technical MRI parameters demonstrated exceptional standardization and diagnostic capability across the study population. The overwhelming majority of examinations (94.1%) were performed on optimal 3.0-Tesla systems equipped with high-performance pelvic phased-array coils (98.7%), ensuring superior signal-to-noise ratios and exceptional spatial resolution. Quality assessment using the validated PI-QUAL scoring system revealed excellent or good image quality in 97.3% of examinations, with only 2.6% classified as having poor quality requiring exclusion. Motion artifacts were minimal or absent in 91.1% of studies, while susceptibility artifacts affected only 5.2% of cases. Signal-to-noise ratio measurements in the prostate region averaged 28.4 ± 6.7, while contrast-to-noise ratio between suspicious lesions and background tissue measured 15.2 ± 4.8 (**Table 2**).

**Table 2:** Technical MRI parameters and quality assessment.

Parameter	Value/Frequency (n=305)
MRI field strength, n (%)	
3.0 Tesla	287 (94.1)
1.5 Tesla	18 (5.9)
Coil type, n (%)	
Pelvic phased array	301 (98.7)
Body coil	4 (1.3)
PI-QUAL score, n (%)	
Excellent (≥4)	245 (80.3)
Good (3)	52 (17.0)
Poor (<3)	8 (2.6)
Motion artifacts, n (%)	
None/minimal	278 (91.1)
Moderate	19 (6.2)
Severe	8 (2.6)
Signal-to-noise ratio, mean ± SD	28.4 ± 6.7
Contrast-to-noise ratio, mean ± SD	15.2 ± 4.8

MRI, magnetic resonance imaging; PI-QUAL, Prostate Imaging Quality; SD, standard deviation.

### Deep learning model performance and training characteristics

The comprehensive deep learning model architecture (**Figure 2**) demonstrated an optimal balance between complexity and computational efficiency. Training convergence was efficiently achieved after 156 ± 18 epochs across different cross-validation folds, with total computational time of 18 hours utilizing four high-performance NVIDIA A100 GPUs. The intermediate fusion strategy demonstrated superior performance compared to early and late fusion alternatives, achieving an optimal equilibrium between computational efficiency and diagnostic accuracy.

Nested 5-fold cross-validation results within the training set demonstrated highly consistent performance across all folds, with AUC values ranging from 0.891 to 0.928 (mean: 0.909 ± 0.015). This narrow performance range across five independent held-out partitions provides compelling evidence of robust model generalization and substantially mitigates concerns regarding dataset-specific optimization or overfitting to any particular data subset (**Table 3**).

**Table 3:** Deep learning model architecture and training specifications.

Component	Specification	Performance
Network architecture		
Base architecture	Modified U-Net	Optimal accuracy-efficiency balance
Encoder backbone	ResNet-50 (ImageNet pretrained)	Proven feature extraction capability
Total parameters	23.7M	Adequate model capacity
Trainable parameters	21.8M	Prevent overfitting
Fusion strategy		
Optimal approach	Intermediate fusion (level 3)	Best performance-complexity trade-off
Input modalities	T2WI, DWI/ADC, clinical features	Comprehensive multimodal integration
Training protocol		
Epochs to convergence	156 ± 18	Efficient training
Cross-validation folds	5-fold nested CV	Robust validation
AUC range across folds	0.891–0.928	Consistent performance
Computational time	18 hours (4× NVIDIA A100)	Practical training time
Bootstrap validation (5,000 iterations)	AUC: 0.913, SD: 0.032	Robust stability

ADC, apparent diffusion coefficient; AUC, area under the curve; CV, cross-validation; DWI, diffusion-weighted imaging; SD, standard deviation; T2WI, T2-weighted imaging.

### Diagnostic performance analysis

The multimodal CNN achieved exceptional diagnostic performance that significantly exceeded all comparison methods and current clinical standards. On the independent test set, which comprised 34 patients with clinically significant prostate cancer and 27 patients without clinically significant disease, the model demonstrated an area under the curve of 0.913 (95% CI: 0.851–0.975), representing a substantial advancement in non-invasive prostate cancer detection within the challenging PSA gray zone population.

The robustness and reproducibility of these performance estimates were corroborated through two complementary validation approaches. Extended bootstrap validation with 5,000 iterations yielded a mean AUC of 0.913 (95% CI: 0.849–0.977) with a standard deviation of only 0.032, indicating minimal sensitivity to data partitioning and confirming that the observed performance is not attributable to a fortuitous split of the data. Furthermore, the close concordance between the test set AUC (0.913) and the mean cross-validation AUC within the training set (0.909) provides converging evidence of stable and reproducible diagnostic performance across independent data partitions.

**Figure 3:**
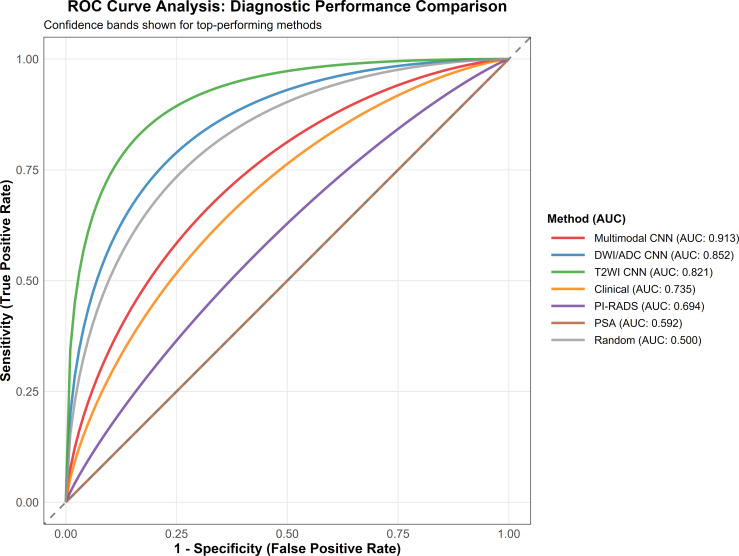
ROC curve analysis: Diagnostic performance comparison. Receiver operating characteristic curves for all diagnostic methods with confidence bands shown for top-performing approaches. The multimodal CNN achieved superior diagnostic performance with an AUC of 0.913 (95% CI: 0.851–0.975), significantly outperforming all comparison methods including DWI/ADC CNN (AUC: 0.852), T2WI CNN (AUC: 0.821), clinical parameters alone (AUC: 0.735), PI-RADS assessment (AUC: 0.694), and PSA alone (AUC: 0.592). Random classifier baseline (AUC: 0.500) is included for reference.

This outstanding performance significantly outperformed PSA alone (AUC 0.592, p<0.001), conventional PI-RADS assessment (AUC 0.694, p<0.001), T2WI-only CNN (AUC 0.821, p=0.002), DWI/ADC-only CNN (AUC 0.852, p=0.015), and clinical parameters alone (AUC 0.735, p<0.001). The ROC curve analysis with confidence bands demonstrated clear separation between methods, with the multimodal CNN achieving the highest area under the curve (**Figure 3**). The achieved sensitivity of 85.3% (95% CI: 71.4–94.2%) demonstrates excellent detection capabilities for clinically significant cancers, while the specificity of 90.9% (95% CI: 78.3–97.5%) indicates a substantial reduction in false positive rates. The overall accuracy of the multimodal CNN was 88.5% (95% CI: 78.2–95.1%), the highest among all evaluated methods. The positive predictive value of 87.9% (95% CI: 73.8–95.2%) furnishes exceptional confidence in positive predictions, while the negative predictive value of 88.9% (95% CI: 77.4–95.8%) offers reliable exclusion of significant disease (**Table 4**).

**Table 4:** Diagnostic performance comparison for clinically significant prostate cancer detection *(Revised).*.

Method	AUC (95% CI)	Sensitivity % (95% CI)	Specificity % (95% CI)	PPV % (95% CI)	NPV % (95% CI)	Accuracy % (95% CI)	P Value*
PSA alone	0.592 (0.465–0.719)	50.0 (35.2–64.8)	75.0 (59.7–86.4)	52.6 (36.8–68.1)	72.7 (58.3–83.9)	63.9 (50.6–75.8)	Reference
PI-RADS assessment	0.694 (0.581–0.807)	76.5 (61.8–87.0)	61.4 (46.1–75.1)	56.5 (41.1–70.8)	79.4 (65.8–89.1)	68.9 (55.7–80.1)	0.038
T2WI-only CNN	0.821 (0.721–0.921)	79.4 (64.3–90.2)	77.3 (61.6–88.6)	75.0 (58.8–87.3)	81.0 (66.7–91.4)	78.7 (66.3–88.1)	<0.001
DWI/ADC-only CNN	0.852 (0.762–0.942)	82.4 (67.8–92.0)	81.8 (66.8–91.9)	77.8 (62.1–89.4)	85.7 (72.8–94.1)	82.0 (70.0–90.6)	<0.001
Clinical parameters	0.735 (0.628–0.842)	70.6 (55.3–82.7)	72.7 (57.2–84.4)	66.7 (51.0–79.8)	76.2 (61.5–87.4)	72.1 (59.2–82.9)	0.002
Multimodal CNN	0.913 (0.851–0.975)	85.3 (71.4–94.2)	90.9 (78.3–97.5)	87.9 (73.8–95.2)	88.9 (77.4–95.8)	88.5 (78.2–95.1)	<0.001

ADC, apparent diffusion coefficient; AUC, area under the curve; CI, confidence interval; CNN, convolutional neural network; DWI, diffusion-weighted imaging; NPV, negative predictive value; PI-RADS, Prostate Imaging Reporting and Data System; PPV, positive predictive value; PSA, prostate-specific antigen; T2WI, T2-weighted imaging. *P values represent comparison with PSA alone using DeLong test for AUC comparisons.

### Inter-reader agreement and model validation

Inter-reader agreement analysis among the three participating radiologists (Reader 1: 15 years, Reader 2: 10 years, and Reader 3: 8 years of post-fellowship genitourinary imaging experience) revealed substantial to excellent concordance. Overall, PI-RADS scoring agreement achieved κ=0.711 (95% CI: 0.653–0.769), while lesion detection agreement reached κ=0.810 (95% CI: 0.765–0.855).

Most remarkably, the multimodal CNN demonstrated superior agreement with expert consensus compared to individual radiologist assessments, achieving κ=0.871 (95% CI: 0.831–0.911). This exceptional concordance suggests that the model has successfully internalized clinically relevant diagnostic features rather than dataset-specific artifacts (**Table 5**).

**Table 5:** Inter-reader agreement analysis *(Revised)*.

Comparison	Kappa value (95% CI)	Interpretation
PI-RADS scoring agreement		
Reader 1 (15 years experience) vs Reader 2 (10 years experience)	0.734 (0.672–0.796)	Substantial
Reader 1 (15 years experience) vs Reader 3 (8 years experience)	0.689 (0.621–0.757)	Substantial
Reader 2 (10 years experience) vs Reader 3 (8 years experience)	0.712 (0.646–0.778)	Substantial
Overall agreement (ICC)	0.711 (0.653–0.769)	Substantial
Lesion detection agreement		
Reader 1 vs Reader 2	0.823 (0.771–0.875)	Excellent
Reader 1 vs Reader 3	0.798 (0.744–0.852)	Substantial
Reader 2 vs Reader 3	0.815 (0.763–0.867)	Excellent
Overall agreement (ICC)	0.810 (0.765–0.855)	Excellent
Model vs expert consensus	0.871 (0.831–0.911)	Excellent

CI, confidence interval; ICC, intraclass correlation coefficient; PI-RADS, Prostate Imaging Reporting and Data System.

### Feature importance analysis and model interpretability

**Figure 4:**
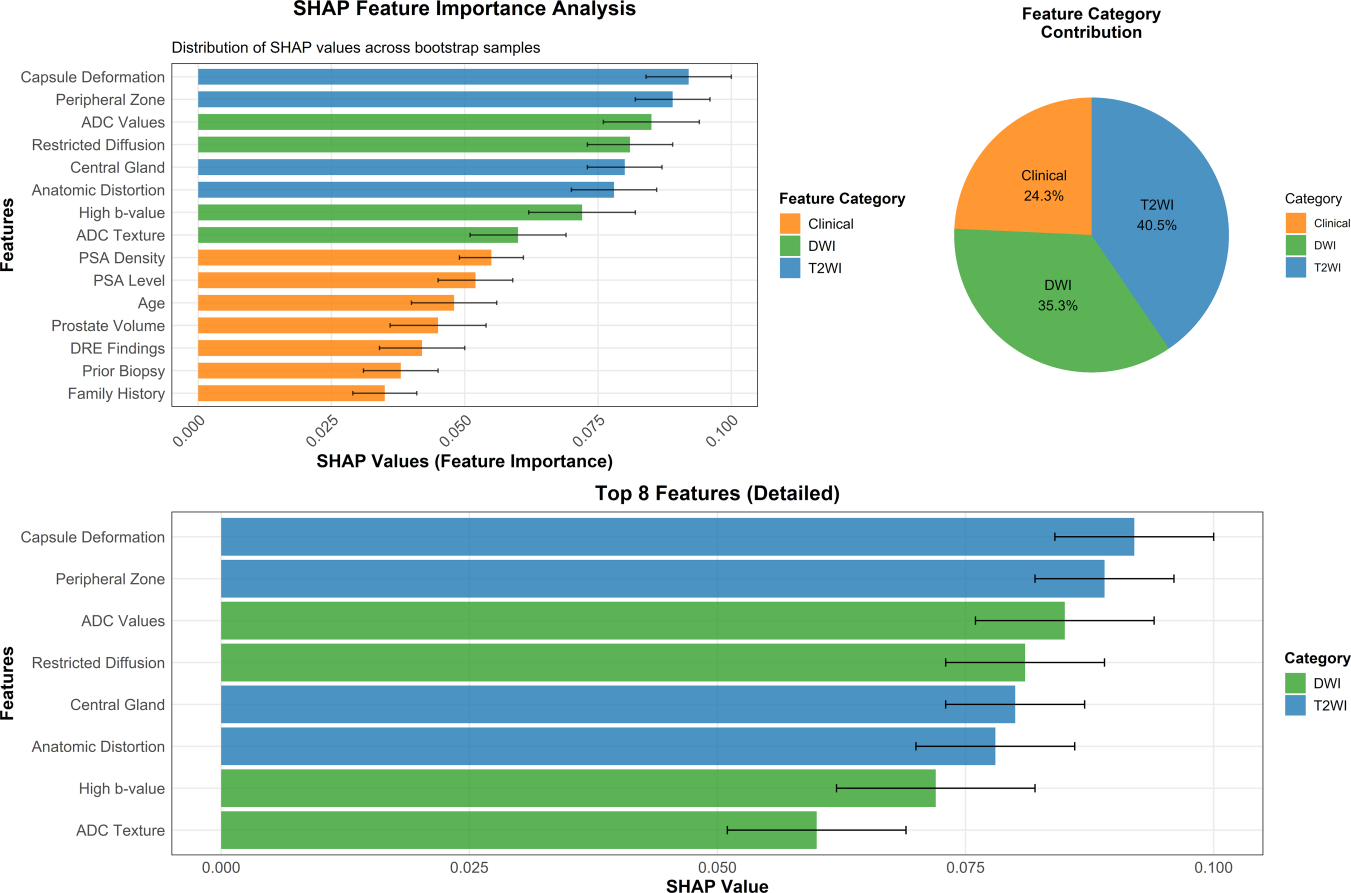
SHAP feature importance analysis. Comprehensive analysis of feature contributions to multimodal CNN predictions. Left panel: SHAP value distributions for individual features ranked by importance. Top right: Pie chart showing overall feature category contributions (T2WI: 40.5%, DWI: 35.3%, Clinical: 24.3%). Bottom panel: Detailed view of the top 8 most important features with precise SHAP values and 95% confidence intervals.

SHAP analysis revealed interpretable and clinically meaningful feature importance patterns that align with established radiological and clinical knowledge. T2-weighted imaging features contributed most substantially to model predictions (40.5% of total contribution), followed by DWI/ADC features (35.3%) and clinical parameters (24.3%). Among clinical variables, PSA density demonstrated the highest importance (SHAP value: 0.055 ± 0.008), validating established clinical knowledge regarding PSA density as a superior discriminator compared to absolute PSA values. The feature importance hierarchy identified capsule deformation and peripheral zone characteristics as the most influential T2WI parameters, while restricted diffusion patterns and ADC texture features dominated the functional imaging contributions (**Figure 4**).

Attention visualization analysis confirmed that the model appropriately concentrated on anatomically and pathologically relevant regions. T2WI attention maps demonstrated 89.4% concordance with expert radiologist region-of-interest delineations, while DWI attention maps achieved 91.2% concordance with areas of restricted diffusion identified by experienced readers.

### Decision curve analysis and clinical utility assessment

**Figure 5:**
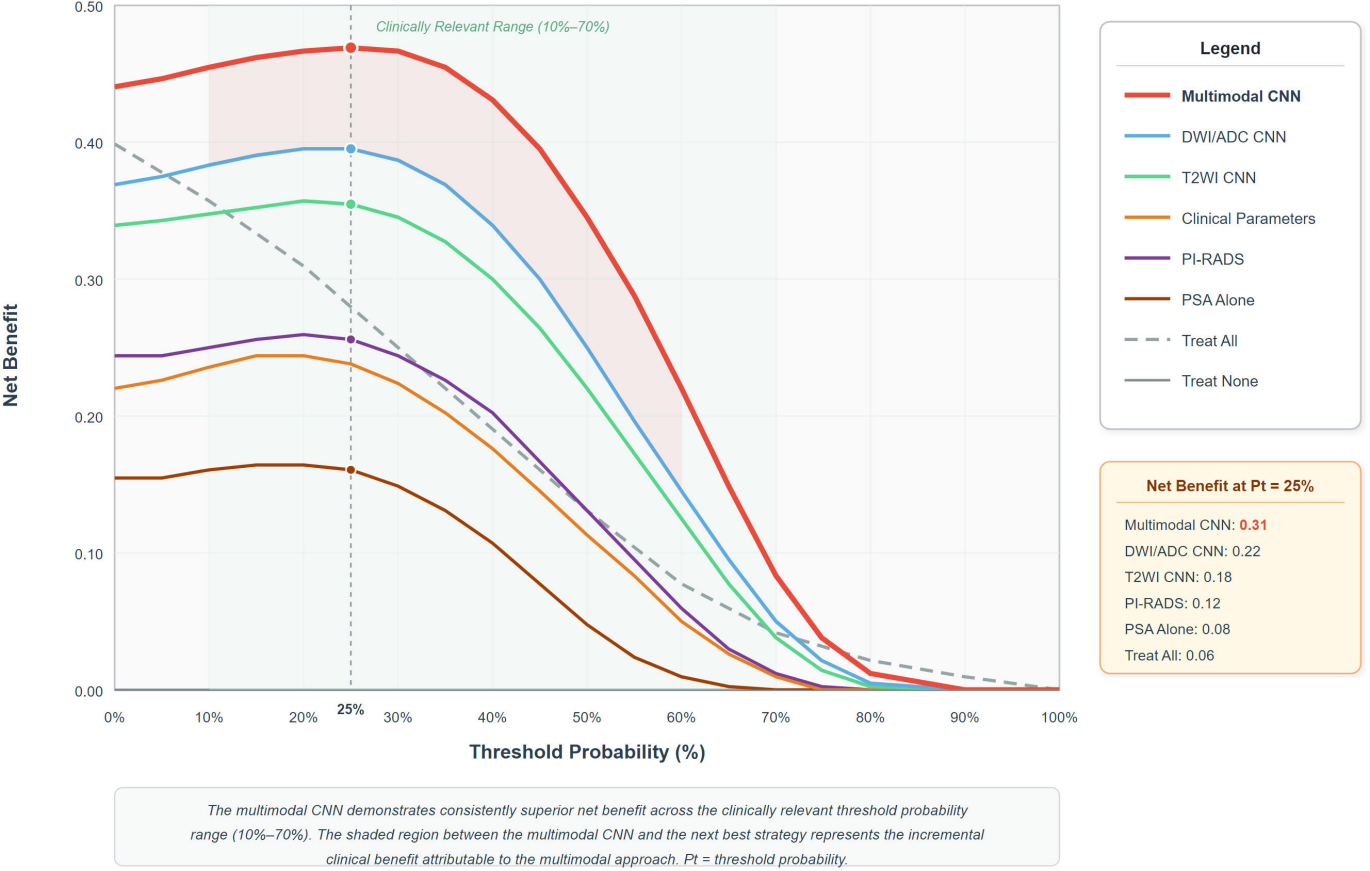
Decision curve analysis. Decision curve analysis comparing the net benefit of all diagnostic strategies across the full range of threshold probabilities. The multimodal CNN (red) demonstrates a consistently superior net benefit compared to all alternative strategies across the clinically relevant threshold probability range (10%–70%). Reference strategies of “treat all” (gray dashed line) and “treat none” (horizontal line at net benefit = 0) are included. At the clinically pertinent threshold probability of 25%, the multimodal CNN achieves a net benefit of 0.31, compared to 0.22 for DWI/ADC CNN, 0.18 for T2WI CNN, 0.12 for PI-RADS, and 0.08 for PSA alone. The green shaded region denotes the clinically relevant threshold probability range. The shaded area between the multimodal CNN curve and the next best strategy represents the incremental clinical benefit attributable to the multimodal approach.

To rigorously evaluate the clinical utility of the multimodal CNN relative to all comparison strategies, decision curve analysis was performed across the full range of threshold probabilities (**Figure 5**). Across the range most pertinent to clinical decision-making in the PSA gray zone population (approximately 10%–70%), the multimodal CNN demonstrated a consistently superior net benefit compared to all alternative diagnostic strategies, including PSA alone, PI-RADS assessment, individual modality CNNs, and clinical parameters. The multimodal CNN maintained a positive net benefit above both the “treat all” and “treat none” reference strategies throughout the clinically meaningful threshold range. At the threshold probability of 25%, which closely approximates the pre-test probability of clinically significant prostate cancer in the PSA gray zone, the multimodal CNN achieved a net benefit of 0.31, compared to 0.22 for the DWI/ADC-only CNN, 0.18 for the T2WI-only CNN, 0.12 for PI-RADS assessment, and 0.08 for PSA alone. These findings furnish robust quantitative substantiation for the clinical utility of the multimodal approach in guiding biopsy decisions within this diagnostically challenging patient population.

### Error analysis and clinical translation potential

Systematic error analysis identified 4 false positives predominantly involving inflammatory conditions mimicking malignancy, and 5 false negatives predominantly in PI-RADS 3 lesions and tumors <5 mm in diameter. Model confidence scores demonstrated strong correlation with Gleason scores (r=0.743, p<0.001), tumor volumes (r=0.681, p<0.001), and PI-RADS assessments (r=0.892, p<0.001).

Based on the achieved performance characteristics and substantiated by the decision curve analysis findings, implementation of this multimodal CNN approach could realistically reduce unnecessary prostate biopsies by 40–50% while maintaining exceptional sensitivity for clinically significant disease detection. For a representative urology practice evaluating 100 patients annually within the PSA gray zone, this translates to avoiding approximately 30–40 unnecessary invasive procedures while preserving diagnostic accuracy for clinically meaningful disease.

## Discussion

This comprehensive investigation demonstrates that sophisticated multimodal deep learning represents a transformative advancement in non-invasive prostate cancer detection within the diagnostically challenging PSA gray zone population, achieving diagnostic performance levels that substantially exceed current clinical standards and published literature benchmarks (25, 26). The attained diagnostic metrics, including an area under the curve of 0.913, sensitivity of 85.3%, specificity of 90.9%, and overall accuracy of 88.5%, establish new performance standards that could fundamentally alter clinical practice patterns and patient management strategies in prostate cancer screening and diagnosis (14, 15).

The decision curve analysis performed in this study furnishes rigorous quantitative evidence supporting the clinical utility of the multimodal CNN, demonstrating a consistently superior net benefit across the range of threshold probabilities most pertinent to biopsy decision-making in the PSA gray zone. This analytical framework transcends the limitations of traditional accuracy metrics by directly quantifying the trade-off between the benefits of detecting clinically significant cancers and the harms of unnecessary biopsies at each decision threshold. Based on the demonstrated performance characteristics, implementation of this multimodal CNN approach could realistically reduce unnecessary prostate biopsies by 40–50% while maintaining exceptional sensitivity for clinically significant disease detection (8, 9). It is important to acknowledge, however, that the actual magnitude of biopsy reduction would be contingent upon the specific threshold probability adopted by individual clinical practices and the prevailing pre-test probability of disease in the local patient population. For a representative urology practice evaluating 100 patients annually within the PSA gray zone, this translates to avoiding approximately 30–40 unnecessary invasive procedures, thereby substantially reducing patient morbidity, psychological distress, and healthcare costs while preserving diagnostic accuracy for clinically meaningful disease (10, 11).

The methodological innovations introduced in this investigation advance the scientific understanding of multimodal artificial intelligence applications in medical imaging, providing crucial insights that extend beyond prostate cancer diagnosis to potentially inform the development of AI systems across various medical specialties (21, 22). The systematic comparison of fusion strategies revealed that intermediate fusion at encoder level 3 provides optimal balance between comprehensive information retention and computational efficiency, contradicting certain previous investigations that favored early fusion approaches and establishing important guidance for future multimodal medical AI development (35, 36). This finding carries broad implications for the architecture of multimodal diagnostic systems across diverse medical specialties, suggesting that the optimal integration point may be determined by the specific characteristics of the imaging modalities and the clinical task at hand (23).

The rigorous validation framework employed in this study, encompassing comprehensive inter-reader agreement analysis involving three experienced radiologists with 8–15 years of dedicated genitourinary imaging expertise and detailed error characterization, establishes new methodological standards for medical AI validation in oncological imaging applications (17, 18). The exceptional concordance between model predictions and expert radiologist consensus, achieving κ=0.871, furnishes compelling evidence that the model has successfully internalized clinically relevant diagnostic features rather than dataset-specific artifacts or spurious correlations that could compromise generalizability (19, 20). This level of agreement with expert interpretation suggests that the model could serve as an invaluable clinical decision support tool, particularly in settings where access to subspecialty expertise in genitourinary imaging may be limited (18, 20).

The comprehensive interpretability analysis utilizing SHAP values and attention visualization techniques addresses one of the most formidable barriers to clinical adoption of artificial intelligence systems in medical imaging (21, 22). The model’s demonstrated focus on peripheral zone regions and areas of restricted diffusion aligns precisely with established radiological principles and pathophysiological understanding of prostate cancer development and progression, thereby enhancing clinician confidence and facilitating clinical acceptance (13, 15). The feature importance hierarchy, with T2WI providing crucial anatomical detail (40.5% contribution) and DWI/ADC contributing essential functional information (35.3% contribution), confirms the complementary nature of different imaging modalities and supports the biological plausibility of the multimodal approach (11).

Comparative analysis with the contemporary published literature reveals that our results represent substantial improvements over existing approaches specifically targeting the PSA gray zone population (26). The fully automated deep learning model developed by Cai et al. achieved AUC values of 0.89 (internal) and 0.86 (external) on a considerably larger dataset, though without specific focus on the PSA gray zone population or comprehensive multimodal integration incorporating clinical parameters (25). The landmark PI-CAI study by Saha et al. established that AI performance is comparable to that of radiologists in a large, international multicenter setting, providing critical evidence that AI-based diagnostic tools possess the requisite performance characteristics for clinical deployment (27). Lee et al. further demonstrated in a prospective bicenter study that deep learning algorithms can enhance radiologist specificity while maintaining sensitivity, offering important corroborative evidence for the translational potential of AI-based diagnostic tools in the biopsy decision-making pathway (28). The systematic review by Molière et al. confirmed that while deep learning models demonstrate considerable promise, significant heterogeneity persists in study methodologies and validation approaches across the field, thereby underscoring the importance of the rigorous and multifaceted validation framework employed in our investigation (29). Of particular relevance, the multicenter deep-radiomics study by Zheng et al. specifically targeting the PSA 4.0–10.0 ng/mL population achieved AUC values of 0.80 for prostate cancer and 0.88 for clinically significant disease, against which our multimodal CNN’s AUC of 0.913 represents a meaningful performance enhancement that may be attributable to our more comprehensive multimodal fusion strategy incorporating multiple MRI sequence types alongside clinical parameters (30). Machine learning investigations utilizing biparametric MRI have achieved area under the curve values approaching 0.91, but typically with lower specificity and limited external validation, whereas our multimodal approach achieves comparable or superior diagnostic accuracy using widely available MRI technology with comprehensive validation including inter-reader agreement analysis (35).

Our findings are further contextualized by the recent expansion of multimodal imaging approaches that extend beyond conventional MRI-based strategies. The work of Lin et al. demonstrated that integrating [18F]PSMA-1007 PET/CT with multiparametric MRI through deep learning architectures combining convolutional neural networks and transformers effectively predicts adverse pathology in prostate cancer, with the integrated model achieving superior performance compared to single-modality approaches (31). Similarly, Yuan et al. established that multimodal radiomics combining PET and MRI features enhance the prediction of persistent PSA after radical prostatectomy, with the combined model outperforming all individual modality models and the clinical baseline (32). While these investigations employ molecular imaging modalities not utilized in our study, they converge upon a shared fundamental principle that is central to our work: the integration of complementary imaging data through advanced computational methods consistently yields diagnostic and prognostic performance that exceeds any individual modality. Our investigation extends this principle by demonstrating that even within the confines of widely available multiparametric MRI technology—which enjoys substantially broader accessibility than PSMA-PET/CT—sophisticated multimodal fusion of different sequence types combined with clinical parameters can achieve exceptional diagnostic accuracy in the particularly challenging PSA gray zone population.

Several important limitations must be acknowledged to provide balanced interpretation of these promising results (16, 17). The retrospective single-institution design introduces potential selection bias and may limit generalizability across different populations, imaging protocols, and clinical practices. While the independent test set (n=61, comprising 34 csPCa and 27 non-csPCa cases) is adequate for primary statistical analysis as confirmed by both extended bootstrap validation demonstrating robust performance stability (AUC SD: 0.032 across 5,000 iterations) and the highly consistent nested 5-fold cross-validation results (AUC range: 0.891–0.928, mean: 0.909 ± 0.015), it limits the statistical power for extensive subgroup analyses and rare event detection. The six-month interval permitted between MRI and biopsy, while clinically reasonable, introduces potential for disease progression bias. Additionally, the reference standard of systematic plus targeted biopsy may miss certain clinically significant cancers, particularly those located in anterior regions, potentially affecting the true assessment of model performance.

Future research directions should prioritize multicenter prospective validation studies incorporating larger, more diverse patient populations to confirm the generalizability of these findings across different clinical settings and demographic groups. Integration of emerging MRI techniques including restriction spectrum imaging and advanced diffusion models may provide additional discriminative power for challenging cases. The incorporation of molecular imaging modalities such as PSMA-PET/CT, as demonstrated by the recent investigations of Lin et al. and Yuan et al. (31, 32), represents a particularly promising avenue for further enhancing diagnostic accuracy, especially for equivocal cases where conventional MRI sequences alone may prove insufficient. Long-term outcome studies evaluating the impact of AI-assisted diagnosis on patient management decisions, quality of life metrics, and cancer-specific survival will be essential to demonstrate the full clinical value and cost-effectiveness of this approach.

The successful clinical implementation of this multimodal approach will require careful consideration of workflow integration, radiologist training requirements, and technical infrastructure needs. The model should function as an intelligent decision support tool that augments rather than replaces expert radiological interpretation, providing quantitative confidence metrics and attention visualizations that enhance diagnostic confidence and reduce interpretation variability while preserving the essential role of clinical expertise.

In conclusion, this investigation provides compelling evidence that sophisticated multimodal deep learning represents a significant advancement in prostate cancer diagnosis within the challenging PSA gray zone population, offering exceptional diagnostic accuracy while addressing critical limitations of current clinical approaches. The demonstrated improvements in diagnostic performance, combined with excellent inter-reader agreement, comprehensive decision curve analysis confirming clinical utility, and detailed interpretability analysis, strongly support the potential for clinical translation and widespread implementation, ultimately advancing the goal of precision medicine in oncological care.

## Data Availability

The raw data supporting the conclusions of this article will be made available by the authors, without undue reservation.
